# The potential of laminar functional MRI in refining the understanding of epilepsy in humans

**DOI:** 10.1093/brain/awaf320

**Published:** 2025-09-03

**Authors:** Fraser Aitken, Joel S Winston, Jonathan O’Muircheartaigh, David W Carmichael

**Affiliations:** Research Department of Imaging Physics and Engineering, School of Biomedical Engineering and Imaging Sciences, King’s College London, London, SE1 7EH, UK; MRC Centre for the Developing Brain, School of Biomedical Engineering and Imaging Sciences, King’s College London, London, SE1 7EH, UK; Research Department of Early Life Imaging, School of Biomedical Engineering and Imaging Sciences, King’s College London, London, SE1 7EH, UK; Department for Forensic and Neurodevelopmental Sciences, Institute of Psychiatry, Psychology and Neuroscience, King’s College London, London, SE5 8AF, UK; London Collaborative Ultra High Field System (LOCUS) London, St Thomas Hospital, London, SE1 7EH, UK; Research Department of Imaging Physics and Engineering, School of Biomedical Engineering and Imaging Sciences, King’s College London, London, SE1 7EH, UK; Kings College Hospital NHS Foundation Trust, London, SE5 9RS, UK; Research Department of Imaging Physics and Engineering, School of Biomedical Engineering and Imaging Sciences, King’s College London, London, SE1 7EH, UK; MRC Centre for the Developing Brain, School of Biomedical Engineering and Imaging Sciences, King’s College London, London, SE1 7EH, UK; Research Department of Early Life Imaging, School of Biomedical Engineering and Imaging Sciences, King’s College London, London, SE1 7EH, UK; Department for Forensic and Neurodevelopmental Sciences, Institute of Psychiatry, Psychology and Neuroscience, King’s College London, London, SE5 8AF, UK; Research Department of Imaging Physics and Engineering, School of Biomedical Engineering and Imaging Sciences, King’s College London, London, SE1 7EH, UK; MRC Centre for the Developing Brain, School of Biomedical Engineering and Imaging Sciences, King’s College London, London, SE1 7EH, UK; Research Department of Early Life Imaging, School of Biomedical Engineering and Imaging Sciences, King’s College London, London, SE1 7EH, UK; Department for Forensic and Neurodevelopmental Sciences, Institute of Psychiatry, Psychology and Neuroscience, King’s College London, London, SE5 8AF, UK; London Collaborative Ultra High Field System (LOCUS) London, St Thomas Hospital, London, SE1 7EH, UK

**Keywords:** cortical layers, ultra-high-resolution fMRI, electrophysiology, focal seizures, generalized seizures

## Abstract

Despite decades of development and clinical application, drug-resistant epilepsy occurs in 25%–30% of patients. One limiting factor in the success of antiseizure medications are challenges in mapping the neural effects of epilepsy drugs to seizure mechanisms in humans.

Most antiseizure medications were developed in animal models and primarily target nano-scale structures like ion channels and receptors. However, they exert their effects and are typically measured in humans at the macro-scale using techniques like EEG and conventional functional MRI (fMRI). This disconnect between the mechanisms of pharmaceutical interventions and the clinical management of epilepsy leaves a critical gap in our understanding. This is because all seizures, even those of a generalized nature, appear to initiate in intermediate scale, local microcircuits and then propagate from that initial ictogenic zone. Invasive electrophysiological recordings in both animal models and humans have shown that one such microcircuit, cortical layers, and more specifically deep cortical layers, play a critical role in seizure generation in both generalized and focal epilepsies, serving as the critical link between nano-scale dysfunctions and the macro-scale activity observed in seizures.

Laminar fMRI, a technique capable of resolving activity across cortical depths, offers a promising avenue to bridge this gap. By providing a non-invasive measure of laminar response alterations in humans, it could complement animal model and electrophysiological findings, offering novel insights into the layer-specific mechanisms of seizure generation and propagation in humans.

This review discusses evidence for this concept, highlighting key findings from animal models and human intracranial recordings in this regard, and details how laminar fMRI may be able to refine our understanding of epilepsy at the microcircuit level. It concludes with a discussion regarding the possible role of laminar fMRI in improving surgical targeting for focal epilepsies, elucidating the mechanistic effects of antiseizure medications, and ultimately, targeting current and future epilepsy treatments.

## Teaser

Aitken *et al.* highlight laminar fMRI as a tool to connect the neural effects of antiseizure medications with the microcircuits that generate seizures in epilepsy. By resolving activity across cortical layers, this approach could improve understanding of seizure mechanisms, leading to more effective treatments and surgical targeting.

## Introduction

Epileptic seizures are generated by dysfunctional neural networks that are characterized by periodic states dominated by excessive and/or hypersynchronous activity, thought to occur due to an imbalance between neuronal excitation and inhibition.^1,2^ However, despite decades of research and development of pharmaceutical and other therapies, we still have significant limitations in treating epilepsy. Antiseizure medications (ASMs) are the first-line treatment and aim to control seizures rather than alter pathology. They can have significant side effects, while being ineffective in up to one-third of all patients with epilepsy.^3-6^ Therefore, this raises an obvious question, namely, what is missing in our understanding of epilepsy that is holding back the development of new and improved therapies that can more successfully treat epilepsy?

The current understanding of epilepsy in humans and indeed its clinical management relies heavily on macro-scale observations mainly from EEG, PET, MRI and functional MRI (fMRI), which capture activity at the level of brain regions (i.e. temporal lobe) or networks.^7-11^ However, it is well established that epilepsy is a multi-scale disorder, with complex, interacting disruptions at the nano-scale (ion channels/neurotransmitters), micro-scale (small groups of neurons/synapses and their connections) and macro-scale (regions/brain networks), that is often only poorly understood.^1,12-14^ The classic example of this disconnect over scales comes from the clinical application of ASMs.

**Figure 1:**
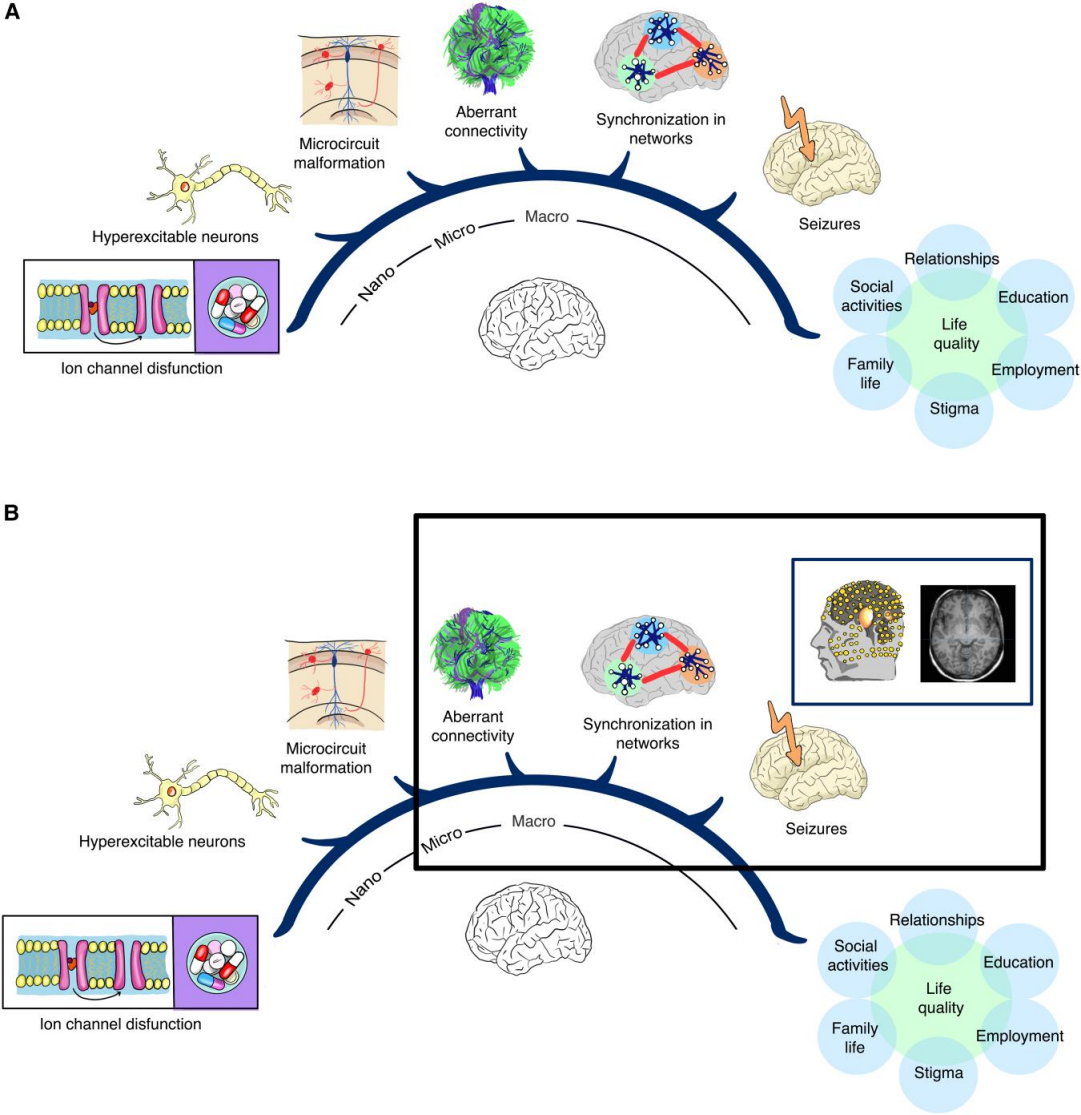
**The multi-scale nature of epilepsy, illustrating the disconnect between nano-scale antiseizure medications and macro-scale human measurements**. Adapted from van den Heuvel *et al*.^20^ (**A**) Broadly, epilepsy is characterized by an imbalance between excitatory and inhibitory signalling in the brain that converts a normally functioning network into a hyperexcitable one. However, while epilepsy is a network disorder, this imbalance can arise due to alterations across a range of scales. For instance, at the nano-scale, changes in ion channel gating can lead to hyperexcitable groups of neurons with alterations at these scales leading to disrupted microcircuit function contributing to seizure generation and spread into macro-scale seizure networks. (**B**) Currently, 30%–40% of patients fail to achieve adequate seizure control with antiseizure medications (ASMs), with success difficult to predict at the point of treatment. One reason for this is the lack of mapping between the mechanisms of ASMs and their clinical applications at the point of treatment. Nearly all ASMs were developed in animal models and primarily target ion channel function, particularly sodium channels, but exert their effects and are measured in humans at the macro-scale with tools like EEG and functional MRI. Measures at this scale are far removed from the proposed targets of ASMs, leading to a situation in which prescription is a trial-and-error process. This situation would benefit from a method to image closer to the nano-scale in humans.

ASMs act primarily on nano-scale targets like ion channels and receptors^3,5,15^ derived in the main from animal model studies,^16^ but exert their therapeutic effects and are measured in humans at the macro-scale using tools like fMRI and EEG.^2,17-19^ Although findings from such modalities provide crucial clinical data, the spatial scales that these imaging tools operate at are far too coarse to directly observe the drug’s effects on their proposed molecular targets (Fig. 1). This leaves a critical gap in our understanding, as the mapping between the impact of ASMs on nano-scale structures and their quantifiable impact on wider brain activity remains difficult to predict at the point of treatment. Essentially, this means that the application of ASMs by clinicians is largely an empirical process, often based upon trial and error within individual patients with epilepsy.^21^

The clinical implications of the disconnect between nano-scale drug mechanisms and macro-scale observations are evident in the variable responses to ASMs, even among patients with epilepsy with seemingly similar epilepsy types given the same type of ASM.^12,21^ Furthermore, the failure of some surgeries to completely eliminate seizures in drug-resistant patients with focal epilepsy^22,23^ highlights our limited understanding of how these multi-scale dysfunctions interact to generate and propagate seizures and the most effective method to reduce seizures. To truly advance epilepsy treatment, research needs to bridge the gap between nano-scale animal studies and macro-scale human research.

Recently, there has been significant discussion within the literature regarding a particular set of findings that may be especially important in connecting our understanding of epilepsy across scales. This debate focuses on the role of alterations in the function of microcircuits in epilepsy.^12,24,25^ Evidence from a range of studies highlighted in this review indicates that these circuits, which are local networks of interconnected neurons, form the bridge between dysfunction in structures like ion channels and the ictogenic network activity captured by modalities like fMRI/EEG that gives rise to full-blown seizures. Importantly, differing pathology at the cellular scale may lead to alterations in microcircuit function, ultimately contributing to seizure generation and propagation across networks.

## Microcircuits: the interface between the nano- and macro-scales

The term microcircuits refers to widely recognized and recurring patterns of neural connectivity and organization within or across brain regions.^26-28^ They are distinguished by their consistent structural organization, comprising specific arrangements of cell types, layers and synaptic connections within defined brain regions. The architecture of microcircuits is intrinsically linked to their functional roles, enabling the execution of specialized computations and processes that underpin various brain functions such as sensory perception, memory consolidation and motor control.^29-31^ This characteristic structure, while establishing a foundation for function, is not static and is also dynamically modulated by experience and ongoing activity.^32^ Furthermore, the evolutionary conservation observed in many of these microcircuits underscores their fundamental significance across diverse species.^33,34^ In short, the concept of microcircuits offers a valuable organizing principle in neuroscience, providing a framework for understanding the intricate mechanisms of brain function and facilitating further investigation into both normal and pathological brain states.

There are several microcircuits in the nervous system. These include thalamocortical circuits,^35,36^ the trisynaptic circuit in the hippocampus,^37,38^ central pattern generators in the spinal cord and brainstem,^39-41^ and the primary subject of this review, the layers of the mammalian cortex.^42-44^ Until relatively recently, it has proven difficult to study the function of such circuits. They operate at scales not easily measurable by standard electrophysiological and imaging techniques, whether invasive or non-invasive. However, technological advances in neuronal imaging and recording techniques have begun to provide detailed descriptions of microcircuit function. The application of state-of-the-art approaches such as intracellular recordings, multi-contact electrodes and optogenetics is beginning to provide unique insight into how microcircuits organize and contribute to generating, propagating and modulating seizure activity (e.g. see Paz and Huguenard^25^).

Within this context, we first introduce the structure and function of cortical layers as a key structural component of microcircuits in neuronal signalling in the healthy cortex, as well as the nature of the vital communication between cortical layers and the thalamus. Next, we discuss evidence for the role of cortical layer dysfunction in generalized genetic epilepsies (GGEs) and their characteristic absence seizures, before considering focal epilepsies, particularly focal cortical dysplasia (FCD). Following this, we outline how laminar fMRI techniques developed in recent cognitive neuroscience work might be useful in translating findings from animal models and invasive methods in humans. Finally, we discuss the potential clinical implications of using laminar fMRI in human epilepsy research, concentrating on improving surgical targeting for focal abnormalities and elucidating the mechanistic effects of ASMs developed via animal models in humans.

## Cortical organization, function and layer-specific electrophysiology: implications for epilepsy

### Cortical organization

**Figure 2:**
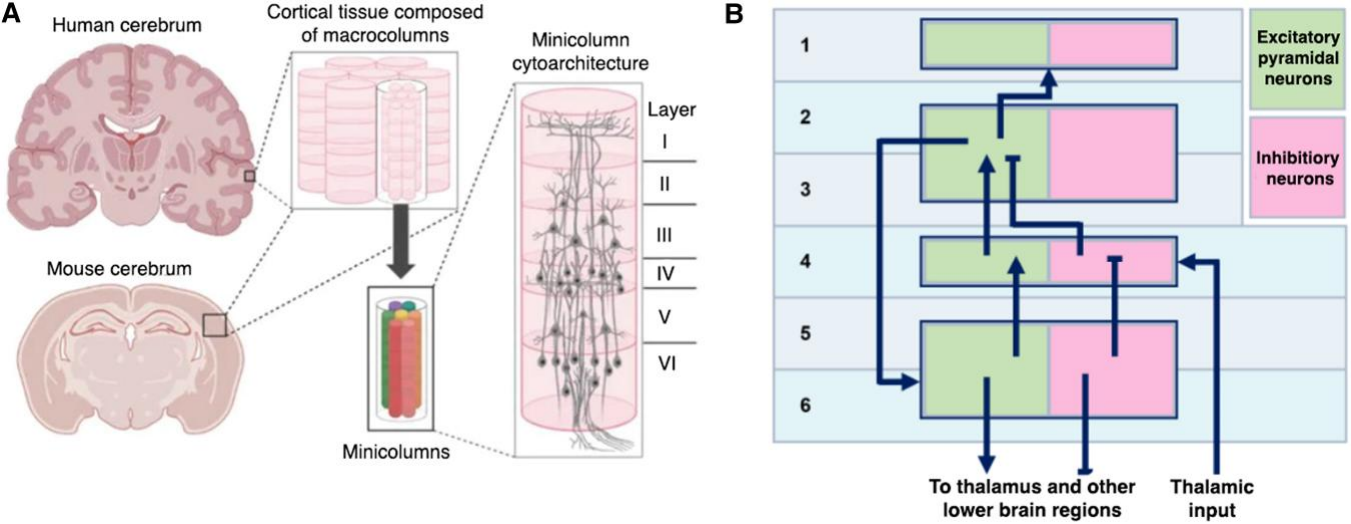
**Cortical organization and laminar microcircuitry. A** shows the organization of the cortex, showing structural similarities between human and mouse brains, illustrating the preservation of lamination across species. The *insets* depict the cortex within the cerebrum, its organization into macrocolumns and minicolumns, and the laminar structure of a cortical column with six distinct layers (1–6). From Brandenburg *et al*.^45^  **B** illustrates a simplified canonical cortical circuit. The thin, solid arrows indicate the direction of information flow and synaptic connections between neurons within and across cortical layers, representing both feedforward processing (e.g. primary sensory input from thalamus largely targets layer 4, which then projects to superficial layers) and feedback processing (e.g. projections from superficial layers back to deep layers or from deep layers to the thalamus). The larger, filled black arrows (e.g. ‘Thalamic Input’, ‘To Thalamus and other lower brain regions’) denote major afferent and efferent pathways, representing overall input and output from the cortical column.

Neurophysiological and neuroanatomical investigations have revealed that the same fundamental organizational principle occurs across the cortex. In all cortical regions, excitatory and inhibitory neurons are arranged into vertical columns and horizontal layers^42,44^ (Fig. 2). Cortical columns, spanning all six layers, function as the brain’s basic processing units, specialized for specific information types.^31,46,47^ Horizontal layers, composed of different densities of excitatory and inhibitory neuron types and connections, orchestrate the hierarchical feedforward and feedback flow of information within columns and layers within cortical areas as well as between columns/layers across the cortex.^48^

The functional principles of the columnar/layered organization of the cortex is often termed the ‘canonical cortical circuit’ and is the cornerstone of cortical function.^30,49-51^ It is characterized by superficial pyramidal neurons receiving feedforward and feedback inputs, deep pyramidal neurons generating cortical output and inhibitory interneurons modulating circuit activity. Central to cortical column/layer function is their interaction with the thalamus. Here, note that while the thalamus and its circuitry is considered to be a separate microcircuit to cortical layers, in the context of certain types of epilepsy and seizures, especially for generalized forms,^52,53^ it plays an important role and can be thought of as a functional part of the same epileptogenic network.

### Thalamocortical interactions

Thalamic nuclei project to specific cortical layers, providing sensory, motor and limbic information.^54-56^ This layer-specific targeting is crucial for information processing. For instance, the primary thalamic nucleus involved in visual processing, i.e. the lateral geniculate nucleus, primarily projects to layer 4 of primary visual cortex, while motor nuclei, i.e. the ventral posterolateral nucleus, target layers 3, 4 and 5 of somatosensory cortex.^57-59^ Thalamocortical and corticothalamic connections form a dynamic loop.^60,61^ Such projections, predominantly glutamatergic, excite cortical neurons, while corticothalamic projections that modulate thalamic activity are primarily GABAergic [a term for anything related to or involving the neurotransmitter gamma-aminobutyric acid (GABA), including neurons that produce and release GABA, synapses that use GABA as a neurotransmitter and receptors that are sensitive to GABA].^62,63^ This interplay is essential for a wide range of brain functions, including perception, attention, arousal and consciousness.^59,61,64^

### Evidence for the role of cortical layer pathology in epilepsy

#### A note on electrophysiological methods

The next sections review evidence of cortical layer dysfunction in seizure generation and spread, drawing from both animal models and human studies of generalized and focal epilepsy. Much of these data come from intracranial electrophysiological recordings (iEEG). To provide context, it is useful to first outline the iEEG recording techniques used in these studies, broadly categorized here as traditional iEEG and multi-contact microelectrode recordings.

Traditional iEEG uses electrodes placed either on the brain’s surface [electrocorticography (ECoG)] or deep within brain structures [stereo-EEG (sEEG)] (see Parvizi and Kastner^65^ for a review of invasive electrophysiological techniques). sEEG electrodes are cylindrical, typically around 2 mm long and 1 mm in diameter, with a surface area of about ∼4 mm^2^, and they penetrate the cortical layers. On the other hand, ECoG electrodes are thin circular disks, with a diameter of several millimetres and a ∼10 mm^2^ surface area, placed directly on the exposed cortex. Both sEEG and ECoG electrodes record signals from a relatively large and varied group of cells. Owing to the sizes of these electrodes and the known neuronal density in the cortex, each electrode records the summed local field potential (LFP) from roughly 500 000 cells. While this provides valuable information about seizure localization, the signal is too broad to decipher the distributed activity patterns among cells within a small area, which is necessary to study cortical layer-specific activity. In contrast, multi-contact microelectrodes offer much higher spatial resolution, making them more appropriate for precisely targeting individual cortical layers.

Instead of having a single contact point, multi-contact electrodes have multiple recording contacts closely spaced along their shaft. The individual recording contacts on these electrodes are much smaller than sEEG electrodes, typically around 10–50 μm in diameter arranged along the electrode shaft with precise spacing, usually between 50 and 200 μm. One type of multi-contact electrode is the laminar electrode, specifically designed to penetrate the cortex perpendicularly, capturing activity across layers with extremely high spatial precision.^66,67^ The development of multi-contact, laminar microelectrodes has enabled the discovery of a consistent pattern of frequency/layer-specific activity termed the spectrolaminar motif.

#### Evidence for layer-specific electrophysiology—a consistent spectrolaminar motif

Scalp EEG provides a valuable overview of brain activity, revealing prominent patterns at specific frequencies,^68^ but emerging evidence suggests a more nuanced picture. Frequency-specific information in EEG appears closely linked to activity within particular brain areas and is influenced by the context of that activity. For example, the occipital lobe, responsible for visual processing, shows strong alpha waves when the subject’s eyes are closed.^69^ Elaborating on these findings, invasive electrophysiological recordings in animals have demonstrated a clear connection between the frequency of LFPs and the cortical layer generating them.^70-74^

In line with this, Mendoza-Halliday *et al*.^75^ investigated whether neuronal activity, as represented in LFPs measured by laminar electrodes, differs systematically between cortical layers and whether this laminar activity pattern is conserved across cortical areas and primate species. Their work revealed a consistent pattern of frequency-specific LFP power gradients across cortical layers, termed the spectrolaminar motif. Specifically, they found that gamma power (50–150 Hz) predominates in superficial layers (2/3), while alpha-beta power (10–30 Hz) is concentrated in deep layers (5/6), with a clear crossover in layer 4. This motif was consistently observed across diverse cortical areas and primate species, highlighting its fundamental nature. Critically, alterations in these background rhythms, as well as the emergence of pathological discharges like rhythmic fast activity, focal slowing and spike-and-wave discharges, are hallmarks of various epilepsy types. These pathological features observed in scalp EEG likely reflect disruptions of the spectrolaminar motif and may therefore map onto the cortical layer dysfunction seen in animal models of epilepsy and the limited amount of human data currently available.

### Data from animal models of absence epilepsy indicate absence seizures originate in deep cortical layers

Absence seizures are a type of generalized onset seizure, frequently associated with idiopathic generalized epilepsies, characterized by brief episodes of unresponsiveness, often accompanied by staring, that occur predominantly in children but can also affect adults.^76,77^ This type of seizure is accompanied by bilateral, rhythmic, highly stereotyped, pathological oscillations (3–4 Hz) evident in scalp EEG recordings called spike-wave discharges (SWDs).^78-83^ The mechanisms of absence seizure onset are still debated, but there is widespread consensus that SWDs originate within the cortico-thalamo-cortical (CTC) system,^84^ and the functioning of this network is essential for the manifestation of complete, bilateral, symmetrical SWD episodes.^85^ Currently, the specific roles and interactions among the different components of the CTC system in the generation and maintenance of SWDs remain a topic of discussion,^24^ with differences observed between rodent models and humans.

Research using mutant rodent models of absence epilepsy has challenged the traditional view of thalamic focus in SWD generation, suggesting instead a cortical origin for these events. Meeren *et al*.^86^ employing non-linear association analysis of ECoG recordings in WAG/Rij (Wistar Auditory Genetic/Rijswijk)^87^ rats, pinpointed the peri-oral region of the somatosensory cortex as the initiation site for SWDs.^86^ This cortical focus exhibited seizure activity that preceded the involvement of other cortical areas and the ventrobasal nuclei of the thalamus by at least 500 ms. Similar but slightly different findings have been observed in the genetic absence epilepsy rat from Strasbourg (GAERS),^88^ where ECoG recordings across multiple cortical and thalamic sites indicated the facial somatosensory cortex as the origin of paroxysmal activity.^89^ In humans, it is important to note that, unsurprisingly, given that these findings are gained from highly inbred rodents, the picture is more variable.

Westmijse *et al*.^90^ used the same non-linear association analysis as used in Meeren *et al*.^86^ for the analysis of pre-SWD → SWD transition periods of MEG, recorded SWD from five medicated children. They discovered focal clusters of highly synchronized activity preceding SWD onset that were consistently located in frontal cortical regions, while sources of the spikes from a train of SWDs were seen in the frontal lateral, central and medial parietal cortices, as confirmed by a beamforming source-localization technique. Importantly, in terms of the current review invasive iEEG techniques are seldom performed in patients suffering from generalized epilepsy, as these patients would not normally be considered for surgical resection. A limited amount of invasive data has been obtained in a small number of patients with a view to targeting deep brain stimulation that demonstrated there may be a patient-specific pattern of seizure initiation between thalamic and frontal regions,^91^ but the recordings made did not resolve cortical layer activity during seizures. However, this level of analysis was performed in one of the rat model studies mentioned above, where invasive electrophysiology studies are possible, and this has also been done to some extent in mouse models.

**Figure 3:**
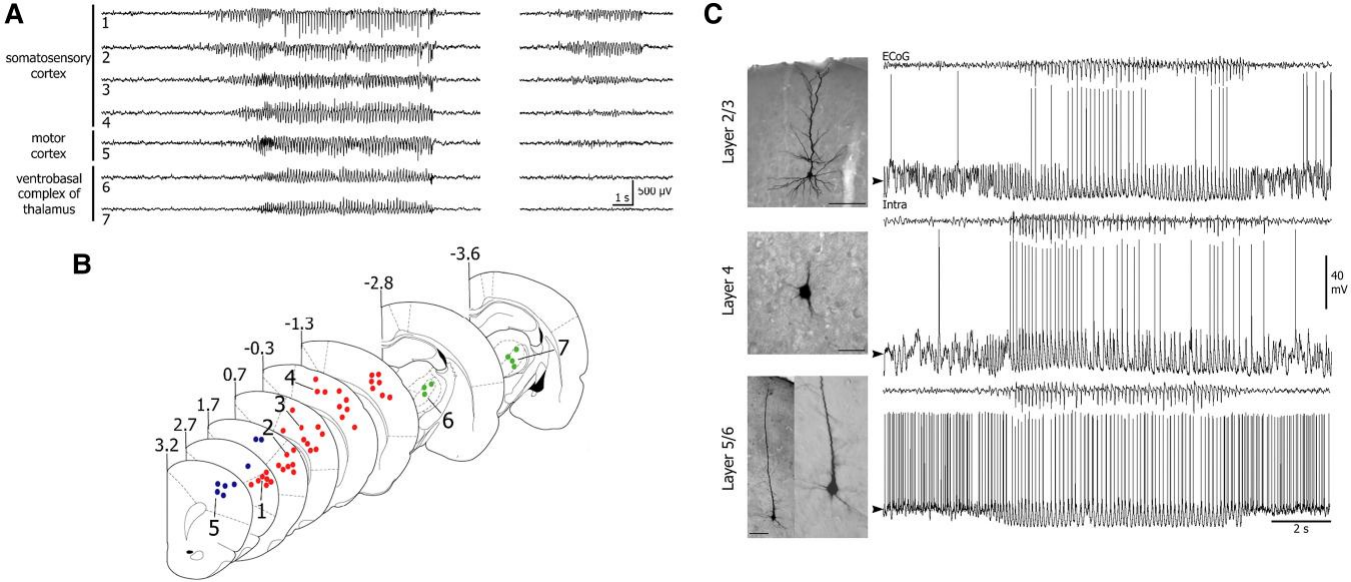
**Spike-wave discharges are initiated within deep layers the facial somatosensory cortex in GAERS.** From Polack *et al*.^89^ (**A**) Simultaneous recordings in freely moving GAERS of local field potentials (LFPs) from the (B) somatosensory and motor cortices, as well as from the ventrobasal complex of thalamus. Spike-wave discharges (SWDs) began in the cortical somatosensory area before propagating to motor cortex and thalamus. (**C**) Simultaneous recordings of intracellular activity and focal electrocorticography (ECoG) showed that during SWDs, all three cell types exhibited rhythmic membrane depolarizations superimposed on a tonic hyperpolarization. Notably, the layer 5/6 neuron displayed a significantly higher interictal and ictal firing rate compared to neurons in the upper layers. GAERS = genetic absence epilepsy rat from Strasbourg.

Polack *et al*.^89^ refined the locality of SWDs provided by ECoG and used intracellular recordings capable of measuring from individual cells to demonstrate that neurons in the deep layers of the facial somatosensory cortex in GAERS exhibited an increase in firing rate shortly before SWD onset (Fig. 3). This contrasted with the lack of such activity in the upper layers of the somatosensory cortex and in the motor cortex. These neurons, which showed a distinctive hyperactivity associated with a membrane depolarization, appeared to lead the firing of distant cortical cells during the epileptic discharge. Consistent with their ictogenic properties, neurons from this focus exhibited interictal and preictal oscillations that were converted into epileptic seizure patterns.

In addition to evidence for the role of cortical layers in the facial somatosensory cortex in rat models of absence seizures, data also suggest changes in the activation of cortical layers in V1 in stargazer mice^92^ during SWDs. Meyer *et al*.^82^ examined the collective activity profiles of individual neurons and surrounding neuropil across all layers in V1 during SWD seizure activity over prolonged periods in stargazer mice.^92^ Using the calcium indicator GCaMP6 with *in vivo* two-photon cellular microscopy and simultaneous ECoG, they showed that most neurons (∼80%) in all cortical layers reduced their firing rates during seizures, whereas a smaller pool (∼15%) increases activity and (∼5%) showed no significant change in activity. Furthermore, ictal participation of identified single-unit activity was not fixed but fluctuated on a flexible timescale from seizure to seizure. Pairwise correlation analysis of calcium activity revealed a lack of synchrony among neurons and neuropil patches in all layers during seizures. These findings are further supported by a recent fMRI study in GAERS, which investigated the responsiveness of visual and somatosensory cortices in GAERS using whole-brain fMRI at 9.4 T. Consistent with Meyer *et al*.,^82^ Stenroos *et al*.^93^ found that whole-brain responses to sensory stimulations were suppressed and spatially hindered during a seizure. These results demonstrate asynchronous suppression of visual cortex during absence seizures, with potential implications for understanding cortical layer function during EEG states of reduced awareness, a fundamental trait of absence seizures.

#### The role of cortical layers in focal epilepsies involving seizures of cortical origin in animals and humans

In refractory focal epilepsy, pre-surgical identification of seizure-onset zones (SOZ) is typically undertaken using non-invasive techniques like assessment of seizure semiology, ictal and interictal EEG recording, structural MRI and PET.^94^ However, if these methods do not locate the SOZ with sufficient confidence, further investigations may be undertaken. If suitable, SOZ characterization may be performed using iEEG implanted for an extended period.^95^

As outlined previously, iEEG allows for the identification of pathological networks involved in seizure propagation, maintenance and spread by measuring LFPs generated by relatively large neuronal populations. There have also been several studies using laminar electrodes in humans, but most use of these electrodes has been to study focal seizure dynamics in animal models and has yielded mixed results. Here, some studies suggest the involvement of all cortical layers,^96^ while others highlight the role of more superficial^97^ or deeper layers^98^ in focal seizure initiation and propagation. One of the very few laminar studies relating to focal epilepsy in humans or at least human tissue was *ex vivo* (using mouse models of glioma and human glioma cell lines) and revealed that gliomas release glutamate, causing hyperexcitability and increased network activity in nearby neurons, particularly in cortical layers 2 and 3.^99^ These inconsistencies across studies are likely due to the inherent differences between focal seizures that are chemically-induced (or via other artificial means)^100^ in animals and naturally occurring focal seizures in humans underscoring the need for further research, particularly studies using laminar electrodes in humans. In this regard, a recent study has provided some ground-breaking data indicating a key role of deep cortical layers in focal seizure generation.

**Figure 4:**
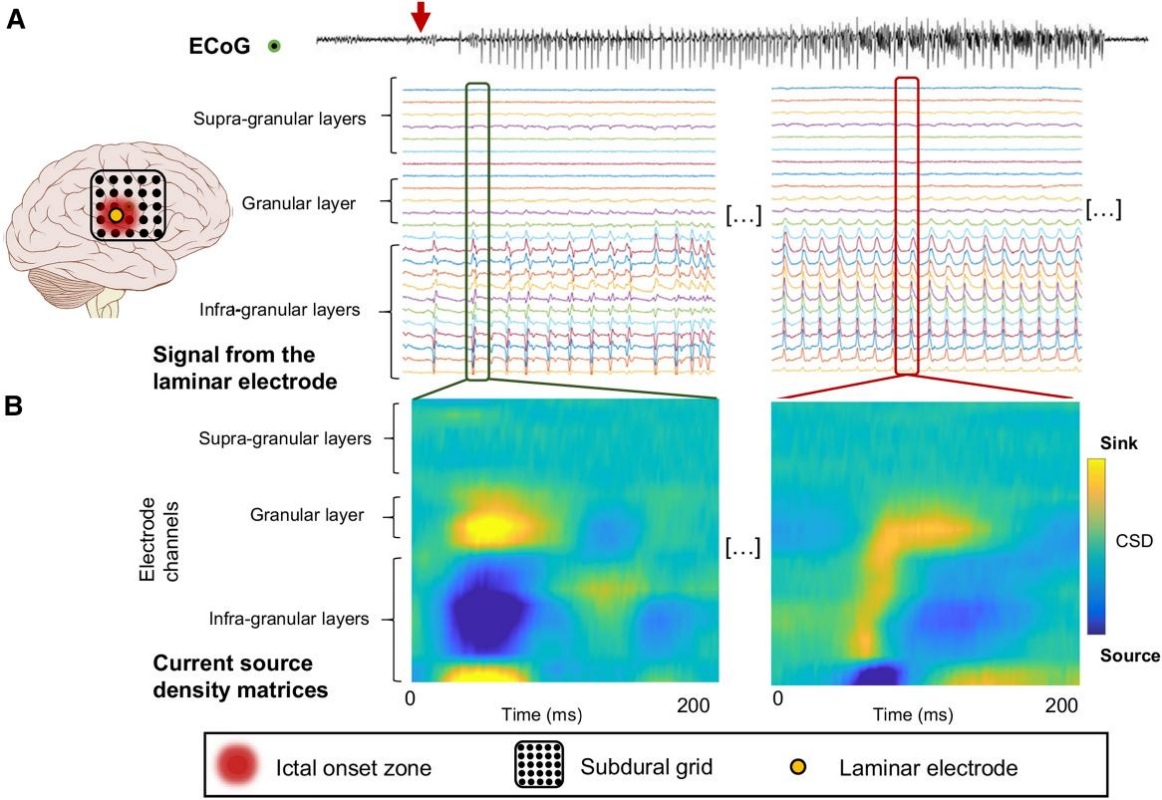
**Human focal seizures initiate in deeper cortical layers.** From Bourdillon *et al*.^100^ (**A**) When the laminar electrode (yellow filled-circle) was positioned within the ictal onset zone (red shading), seizure onset (red arrow) was observed simultaneously with adjacent electrocorticography (ECoG) electrodes, indicating accurate capture of the seizure’s origin. (**B**) Initially, ictal activities were localized to the granular and infragranular layers, with current sources (blue plots) and sinks demonstrating specific current flow patterns; specifically, sinks were found in the adjacent granular cortex and the deepest infragranular cortex. CSD = current source density.

Bourdillon *et al*.^100^ used ECoG to identify the general SOZ and then laminar electrodes to simultaneously record activity across the six layers of the neocortex during focal seizures in humans in the identified region. This study examined 30 seizures recorded from 10 individuals (two females, eight males) with intractable focal epilepsy. These patients presented with diverse epilepsy aetiologies, including FCD, mesial temporal epilepsy, and a brain tumour in one case. Additionally, different neocortical regions were affected across these patients, highlighting the variability in seizure origin in focal epilepsy. The key finding was a clear distinction in the cortical layers involved in seizure initiation versus propagation. To assess seizure initiation, 17 laminar electrodes were implanted in total across the 10 individuals; among these, five were strategically placed within the identified SOZ, with the remainder situated outside the SOZ. This method showed that ictal discharges originated primarily from the deeper granular and infragranular layers (Fig. 4). This suggests that intrinsic properties within these deeper layers play a crucial role in generating seizures. Interestingly, the deepest layer (layer 6) exhibited unique activity patterns, potentially linked to its specialized structure in the human cortex. In contrast, seizure propagation, observed in recordings from electrodes outside the SOZ, involved the superficial supra-granular layers, with activity extending into deeper layers as the seizure progressed. This implies a distinct pathway for seizure spread, relying on connections within the superficial cortex. These findings, observed across different epilepsy aetiologies and brain regions, provide a framework for understanding the cortical microcircuitry underlying seizure generation and propagation, with potential implications for improved seizure localization, surgical interventions and neuromodulation therapies.

In summary, the covered studies, both in animal models and humans, highlight the crucial role of cortical layers in both generalized and focal seizure activity. Animal models of absence epilepsy consistently show seizure initiation in deep cortical layers, a finding supported by both electrophysiological recordings, *ex vivo* and fMRI studies in animals. In humans, recent research using high-resolution, laminar electrode studies has provided evidence that specific cortical layers play a role in focal seizures involving differing epileptogenic pathology, demonstrating that focal seizures originate in deeper layers and then propagate to superficial layers, albeit from one study. Together, this research suggests distinct layer-specific mechanisms and pathways for seizure generation and spread within the cortex. Crucially, while the outlined literature supports the idea that laminar fMRI may be useful in translating these findings to humans, the majority of evidence for the role of cortical layers in seizures arises from electrophysiological recordings. This necessitates a discussion of the link between electrophysiological recordings and fMRI measurements as well as other modalities mentioned in this review, because this is key to the validity of laminar recordings as markers of epileptogenicity in the wider epilepsy literature. In addition, to provide a clear context for the capabilities and limitations of laminar fMRI compared to other established techniques, we have outlined a comparative overview in Supplementary Table 1.

#### The relationship between electrophysiological recordings and fMRI measurements

Motivated by the clinical need to relate invasive electrophysiological findings in animal models to non-invasive measurements in humans, researchers have sought to establish a clearer link between the electrophysiological activity seen in epilepsy and fMRI responses. In terms of scalp EEG, research has been broadly successful in characterizing the relationship between recordings and fMRI responses in both healthy cortex and epilepsy patients.^101,102^ In addition, recent research has demonstrated the feasibility of relating EEG recordings to layer-specific fMRI measurements. For example, Scheeringa *et al*.^103^ used simultaneous EEG and high-resolution fMRI to demonstrate distinct relationships between specific EEG frequency bands and activity in different cortical layers. In a visual attention task, they found gamma-band EEG power correlated positively with the blood oxygen level-dependent (BOLD) signal in superficial cortical layers, while beta-power showed a negative correlation with deep layer BOLD, and alpha-power with both deep and superficial layers. Of course, establishing a similar relationship between iEEG data and fMRI is a more complex logistical challenge, but in a small number of patients undergoing intracranial recordings, simultaneous ECoG and fMRI recordings have been made and provided evidence of a general relationship.

Carmichael *et al*.^104^ investigated the haemodynamic correlates of intracranial interictal epileptic discharges (IEDs)^105^ in three patients with focal epilepsy. Using these methods, the researchers were able to map BOLD changes associated with IEDs, revealing both local and distant haemodynamic changes. The study also focused on activity in the somatosensory cortex during finger tapping and at rest. They reported a pattern of fMRI responses during evoked activity in the motor cortex, where BOLD was positively correlated with gamma band activity. Conversely, at rest, BOLD was most strongly negatively correlated with beta band activity. Importantly, the best predictor of BOLD was a principal component analysis of the iEEG, indicating that multiple spectral components likely contribute to the fMRI signal. While the spatial resolution of the fMRI data in this study was insufficient to demonstrate a localized layer-specific response, it provided evidence suggesting that electrophysiological signatures known to exhibit layer-specific features may be detectable as layer-specific fMRI responses.

Beyond the general correlations between fMRI and electrophysiology, the advent of simultaneous EEG-fMRI at ultra-high magnetic field strengths (e.g. 7 T) has the potential to further aid the investigation of epilepsy microcircuitry. While the Scheeringa *et al*.^103^ study demonstrated the feasibility of correlating EEG frequency bands with laminar fMRI signals at lower field strengths, as discussed, 7 T offers significantly improved signal-to-noise ratio (SNR) and enhanced spatial resolution for fMRI. This boost in fMRI signal quality is particularly beneficial for resolving the subtle, layer-specific haemodynamic changes that laminar fMRI aim to detect.

In clinical terms, integrating the high temporal resolution of EEG with the enhanced spatial specificity/resolution of 7 T laminar fMRI would provide a powerful tool to precisely localize and characterize epileptiform activity, such as interictal spikes and high-frequency oscillations, within specific cortical layers. While the technical complexities of conducting simultaneous EEG at 7 T are substantial,^106,107^ including challenges with increased artefacts from EEG hardware and heightened safety considerations due to stronger magnetic fields, ongoing methodological advancements and dedicated hardware development are rapidly making this combined approach more safely applicable and robust for human epilepsy studies. This novel multimodal approach could be instrumental in bridging the gap between macro-scale network dysfunction and the underlying layer-specific neurophysiology in human epilepsy.

In summary, laminar fMRI, with its capacity to resolve activity across cortical depths, offers immense potential for non-invasive human studies, particularly in investigating layer-specific dynamics in individuals with epilepsy. This could improve our understanding of microcircuit abnormalities and how they map onto treatment response, ultimately informing the development of more targeted epilepsy treatments. A key area of future investigation will need to be to understand the relationship between interictal and ictal laminar functional abnormalities because of the practical limitations of imaging during many types of seizures.

### Laminar fMRI

Laminar fMRI is a technique that aims to provide more detailed information about brain activity by analysing signals at different depths within the cerebral cortex.^108,109^ This method operates under an assumption that the cortical depths from which it acquires signals correspond anatomically to the depth of human cortical layers.^110^ By analysing activity at different cortical depths, laminar fMRI has provided insights into the precise neural mechanisms underlying various cognitive processes involving feedforward and feedback signalling, such as perception,^111-114^ attention^115^ and working memory.^116^ However, while laminar fMRI has so far provided data about the neural mechanisms underpinning these phenomena, and there has been in-depth theoretical discussion about its potential use in neuro-cognitive disorders such as psychosis,^117^ it has not yet been established in the study of neurological disorders. In recent years, there have been a range of developments that might now facilitate this application.

#### Laminar fMRI to study epilepsy: why now?

**Figure 5:**
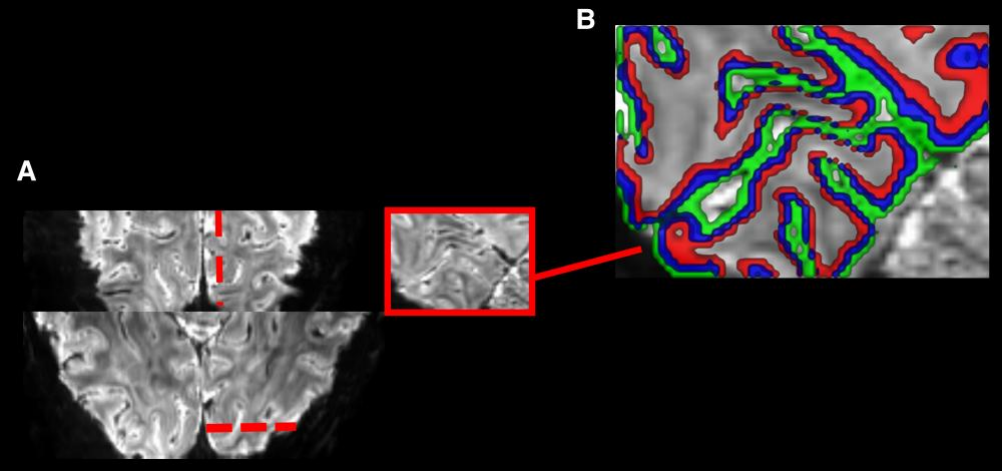
**High resolution functional MRI image of visual cortex and associated extracted layer depths.** Data and image adapted from Aitken *et al*.^121^ (**A**) The image acquired on a Siemens Magnetom 7 T MRI system with a single-channel head coil for localized transmission with a 32-channel head coil insert for reception (Nova Medical) at the Wellcome Centre for Integrative Neuroimaging (University of Oxford), is a T2*-weighted 3D gradient-echo electroplanar imaging sequence (volume acquisition time = 3583 ms, repetition time = 74.65 ms, echo time = 29.75 ms, voxel size 0.8 × 0.8 × 0.8 mm, 16° flip angle, field of view 192 × 192 × 38.4 mm, in-plane generalized autocalibrating partial parallel acquisition (GRAPPA) acceleration factor 4, in-plane partial Fourier 6/8, echo spacing 1.27 ms). (**B**) Deep, middle and superficial cortical layers are indicated in the coloured ribbons. The main image and *inset* are sagittal reformats, which accurately correspond to the highlighted occipital region, with the dotted lines on the axial and coronal images indicating the corresponding location in appropriate terms (*inset*).

Several key advances have paved the way for laminar fMRI methods developed in cognitive neuroscience to now be ready for clinical translation, particularly in the study of epilepsy. First, and perhaps most obviously, is the increasing number of ultra-high field MRI machines with B_0_ field strengths of 7 T and above available in clinical research settings. Field strengths of this level provide the required SNR for acquiring measurable signals from submillimetre size voxels necessary for inferring activity at the cortical depths approximating anatomically to cortical layers^118-120^ (Fig. 5). As access to these scanners has increased, an increasing number of research groups have improved readout and fMRI contrast strategies involving BOLD and non-BOLD techniques like cerebral blood volume related contrasts, especially vascular space occupancy (VASO).^122-125^ This has significantly improved the spatial specificity and contrast weighting necessary for accurate laminar analysis across wider areas of the brain.^109,122^ Improved spatial specificity will likely prove essential for detecting subtle epileptogenic functional alterations caused by structural abnormalities of unclear or unknown location, as is often the case. Finally, a growing understanding of the distinct roles of cortical layers in cognitive processes in humans provides a framework for interpreting how pathological activity in patients with epilepsy might disrupt these intricate networks. By capitalizing on these methodological and conceptual advances, laminar fMRI is poised to reveal the layer-specific signatures of epileptogenicity, potentially leading to a deeper understanding of seizure generation and propagation and ultimately to more effective diagnostic and therapeutic strategies.

#### Isolating markers of epileptogenicity in the laminar fMRI signals

To identify a laminar fMRI marker of epileptogenicity, it is logical to first identify the local signatures of such activity in cortical layers within a specific region where seizures are thought to be generated, or at least the zone that is generally spatially congruent. As outlined, deep cortical layers appear to play an especially important role in the generation of seizures in both GGE-related seizures and in focal seizures of cortical origin. The fact that deep layers appear to be key to epileptogenesis presents an interesting opportunity to incorporate existing laminar fMRI experimental paradigms from cognitive neuroscience that have isolated deep layer activity into the study of epilepsy to explore this initial working hypothesis.

#### Changes in V1 deep layer activity in photosensitive GGE patients as measured by laminar fMRI

This hypothesis is supported by findings from the outlined animal models and human studies that implicate deep cortical layers in the initiation of seizures in various types of epilepsy, including both generalized^82,89,126^ and focal^100^ epilepsies. As highlighted in this review, while animal models suggest absence seizure initiation in somatosensory regions,^86,89^ these data are obtained from highly inbred rodents, and human data are more heterogeneous.^90^ This makes the examination of layers of the visual cortex, particularly in photosensitive epilepsy, a more constrained starting point. Moreover, the high prevalence of photosensitivity in idiopathic generalized epilepsy (IGE), especially in juvenile myoclonic epilepsy (JME),^127,128^ further strengthens the rationale for investigating the visual cortex. In addition, photosensitivity can be mapped onto a laminar circuit view where the expected attenuation of the cortical response to a repetitive stimulus is abnormal. Broadly, this could be conceptualized as a reduced adaption/inhibitory process that should be mediated by deep cortical layers. Therefore, measuring deep layer responses that relate to the expectation of a stimulus could be used to test this hypothesis and provide conceptual validation of the ability of layer fMRI to measure altered circuit properties at this scale.

The next section outlines examples of laminar fMRI experimental design that have successfully manipulated deep layer activity in early visual cortex. Note that the presented studies are not intended to be an exhaustive list but rather illustrative examples of the types of experiments that could be applied to study laminar activity in photosensitive patients.

#### Manipulating deep layer activity in visual cortex using previous laminar fMRI paradigms

Laminar fMRI has provided important information regarding the functional roles of cortical layers in humans. Consistent with animal data in the area,^31,48^ an increasing number of laminar fMRI studies have indicated feedback signals are largely segregated from feedforward signals within each cortical area. Specifically, bottom-up signals predominantly flow from superficial layers 2/3 to the granular layer 4 of downstream regions, and feedback arises from the deep layers 5/6 and targets agranular layers 1 and 5/6 of upstream regions.^51^ Crucially for the study of epilepsy, given the apparent role of deep layers in seizure initiation, previous laminar fMRI studies have shown that it is possible to successfully experimentally manipulate and, in some cases, isolate deep layer from middle and superficial layer activity in visual cortex via cognitive neuroscience paradigms designed for this purpose.

One method to manipulate layers activity used in visual perception studies is to induce visual illusions by presenting ambiguous or conflicting stimuli that the visual system attempts to resolve based on prior experience. Based on these ideas, Kok *et al*.^113^ presented the Kanizsa illusion, which is characterized by the perception of an illusory triangle despite the absence of bottom-up visual evidence in those regions of the image. Using ultra-high-field fMRI, results showed distinct laminar activation patterns in primary visual cortex (V1) that differed between conditions. During trials where the shapes were positioned so that they did not induce the Kanizsa illusion, all cortical layers were activated. Conversely, when the same shapes were arranged to produce the Kanizsa illusion, the perception of the illusion activated the deep layers.

The distinct roles of cortical layers in visual processing, particularly the contributions of deep and superficial layers to top-down modulation, are an ongoing topic of discussion in laminar fMRI research. While some studies^113,116^ report involvement of both layer depths in working memory/attention, potentially reflecting feedback processing related to prior experience, it is crucial to disentangle the specific contributions of working memory *per se* from the sustained attentional/working memory demands inherent in many such tasks, and in some cases, this has been shown to be possible.

**Figure 6:**
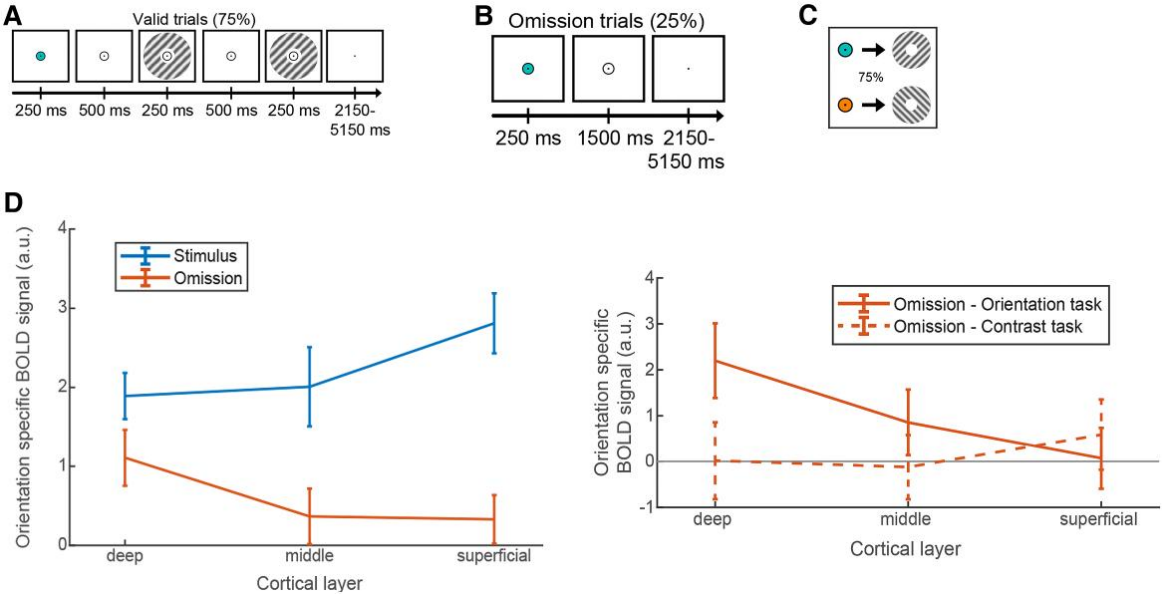
**Experimental paradigm designed to manipulate deep layer activity and results.** Image taken from Aitken *et al*.^121^ (**A**) A coloured dot predicted grating orientation (75% accuracy). Participants then judged either the orientation or contrast difference between two gratings. (**B**) Cue made of a blue/orange circle predicted grating orientation (75% accuracy). (**C**) On 25% of trials, gratings were absent, creating an expectation without visual input. Results showed (**D**) primary visual cortex (V1) activity patterns differed significantly between expected (but omitted) and presented gratings. Expecting a grating activated only the deep layers of V1, not middle or superficial layers. Whereas, when gratings were presented regardless of task, all layers of V1 (deep, middle and superficial) were activated. BOLD = blood oxygen level-dependent.

Aitken *et al*.^121^ (Fig. 6) sought to address the attention/working memory/prior experience ambiguity by employing a paradigm designed to isolate expectation signals. The experimental paradigm started with a coloured dot cue that predicted the orientation of a subsequent grating with 75% validity. In 75% of trials, a set of gratings was then presented, the first of which showed the expected orientation and the second differed slightly in orientation and contrast. In separate experimental runs, participants discriminated either the orientation or the contrast difference between the gratings. In both runs, the colour of the fixation circle (cyan or orange) predicted the orientation of the subsequent grating stimulus (45° or 135°) with 75% validity. Crucially, the gratings were omitted in the remaining 25% of trials. On these trials, there was an expectation of a particular visual stimulus but no actual visual input. These ‘expected but omitted trials’ were crucial because, in the absence of a visual stimulus, any observed laminar activations could only reflect feedback expectation signals, thereby isolating them from attention or other stimulus-driven activity. Results showed that when stimuli were actually presented, activity was seen in deep, middle and superficial layers. This was seen in both types of experimental run. However, crucially, during the ‘expected but omitted’ trials in orientation runs, activity was observed only in the deeper layers, not in middle or superficial layers. Although speculative, this result might be particularly relevant for epilepsy research as it might not only provide an insight into the possible translatability of animal studies, indicating the role of deep layers in seizure generation, but also offers a potential marker of epileptogenicity in studies that examine wider areas of the brain without a task, such as in resting state, connectome-level studies.

#### Connectome analysis detects network-scale functional abnormalities in focal and generalized epilepsies

While task-based laminar fMRI paradigms can reveal layer-specific abnormalities within a predefined region, their utility in the study of focal epilepsies is limited by the inherent variability of seizure foci. Because these paradigms are designed to activate specific areas *a priori* by their nature, they cannot measure subtle abnormalities in other locations. Resting state fMRI,^129^ which measures intrinsic brain activity without a task, offers a more adaptable approach for investigating these epilepsies. In addition, resting state laminar EEG-fMRI could incorporate a marker of epileptogenicity. Examining layer-specific activity and connectivity makes it possible to uncover functional abnormalities associated with the epileptogenic zone. The key initial clinical value of this can be to find brain areas with functional pathology, even when they are not identified on structural MRI, thereby providing targets for surgery or sEEG.^18,130,131^ This approach also facilitates a more comprehensive exploration of the pathological cortical microcircuitry of SOZs. For example, EEG-fMRI often detects networks^7^ where a refined laminar resolution should enable clear identification of its structure in terms of directed connectivity and therefore identification of key network nodes. Furthermore, while connectome-wide disruptions at the laminar scale may be valuable for isolating focal abnormalities, they can also be used to investigate and refine network-wide alterations in generalized epilepsies, where the directed nature within the network has been examined.^132^ This broader application could provide more refined insights into areas of study like the effects of ASMs on network activity, a common focus of pharmaco-fMRI research. However, this approach does have a number of challenges.

Until recently, laminar fMRI studies were mainly confined to individual sensory cortices and employed block-design experiments due to technical limitations or trade-offs in achieving both high spatial resolution and sensitivity across wider areas of the brain (see Huber *et al*.^109^ for an excellent discussion of the following issues). Perhaps the main reason for this can be considered to be that traditional generalized epilepsy-BOLD fMRI used in most laminar fMRI paradigms, while sensitive, suffers from spatial blurring due to strong signals arising from large draining veins, particularly affecting superficial layers. Essentially, this introduces a bias towards these upper layers during analysis. However, advancements in fMRI techniques, such as the use of cerebral blood volume-sensitive contrasts (CBV), like VASO that nulls the impact of large veins and improved data acquisition strategies, have enabled whole-brain coverage at high resolution with acceptable sensitivity. These advancements now allow for the investigation of layer-specific activity and connectivity across the entire brain, opening up new possibilities for probing disruptions in laminar connectivity caused by focal abnormalities across networks even if the location is unclear or unknown.

**Figure 7:**
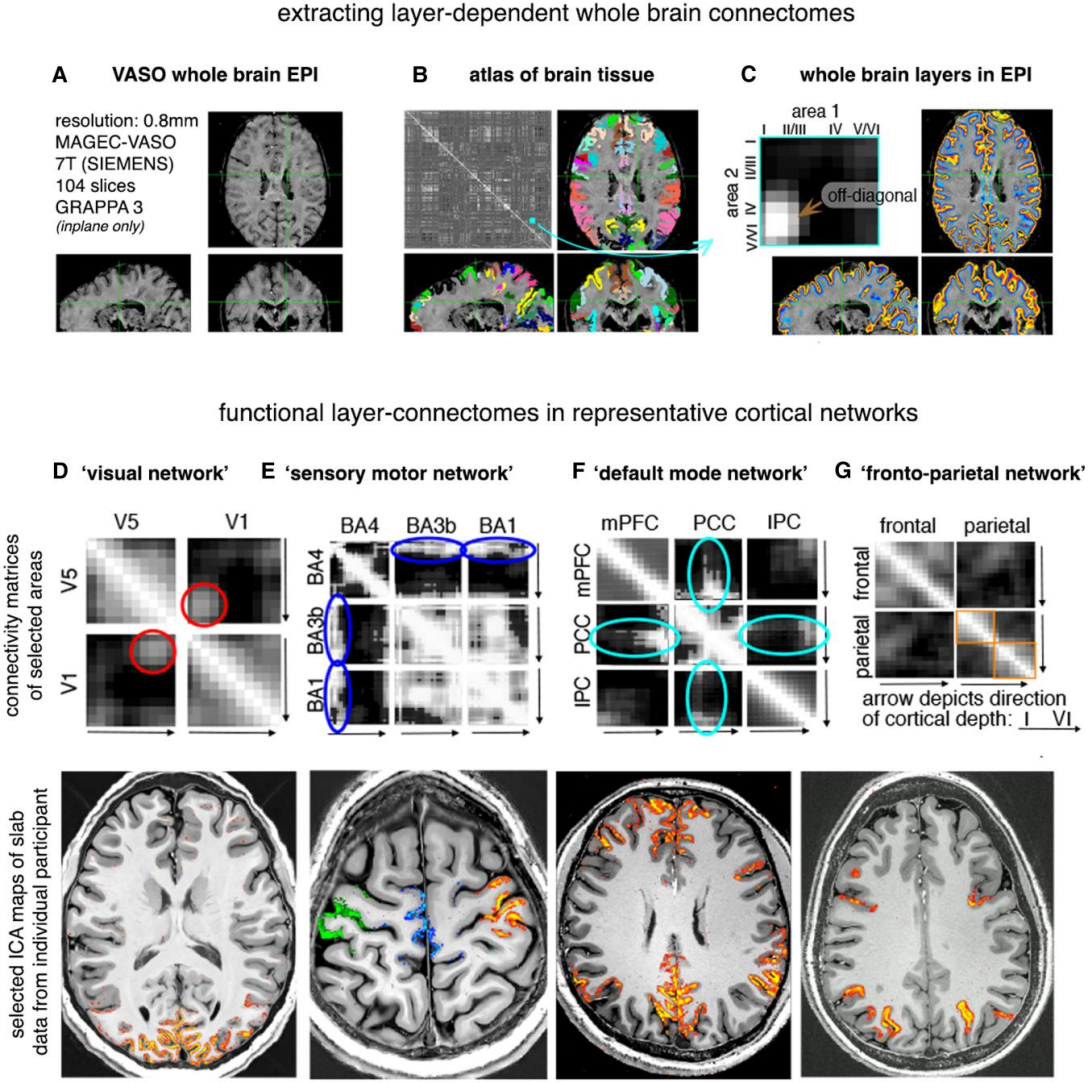
**Whole brain connectome/networks analysis in networks associated with epilepsy is plausible.** Image taken from Huber *et al.*^109^  **A** provides the parameters/quality of raw functional MRI (fMRI) data, with 0.8 mm resolution with vascular space occupancy (VASO), providing excellent contrast for segmentation across the whole brain. **B** explains how functional connectivity matrices are generated by correlating activity between different brain areas, using an atlas for parcellation. **C** highlights the added dimension of layer-specificity, where each node in the matrix now represents connections between specific layers of different areas, allowing for analysis of feedforward and feedback pathways. **D**–**G** provide examples of layer-dependent connectivity in various networks relevant to epilepsy: the visual network (**D**) showing feedback connections between V1 and V5; the sensory motor network (**E**) with expected input patterns to motor cortex; the default mode network (**F**) suggesting a central role for the posterior cingulate cortex (PCC); and the fronto-parietal network (**G**) with distinct within- and between-layer connectivity. This technique allows for detailed examination of how different brain regions and layers interact, offering valuable insights into healthy brain function and potential disruptions caused by epilepsy or lesions like focal cortical dysplasia (FCD) or those seen in the generalized epilepsies linked to genetic causes.

Perhaps the best example of the plausibility of using CBV methods to measure epileptogenic driven alterations in laminar signals across established brain networks comes from Huber *et al*.^109^ (Fig. 7). This study employed VASO and layer-dependent connectome mapping to investigate laminar connectivity across several established brain networks. Their findings supported the canonical hierarchical model in the visual system, indicating top-down feedback flowing to superficial layers in V1 and bottom-up feedforward input to V5/human motion complex (hMT+) middle/deeper layers. The sensorimotor network analysis revealed sensory input predominantly targeting superficial layers of the primary motor cortex. Within the default mode network (DMN), the posterior cingulate cortex emerged as a central hub, exhibiting strong middle-layer connectivity. Lastly, the fronto-parietal network displayed functional segregation, with strong within-layer connectivity but weak between-layer connectivity in superficial and deeper layers. These results highlight the utility of laminar fMRI in deciphering directional connectivity and hierarchical organization, offering crucial insights into the functional specialization of cortical layers within brain networks with distinct implications for detecting layer activity changes across the brain in patients with epilepsy.

### The clinical utility of laminar fMRI

The ability of laminar fMRI in conjunction with suitable experimental paradigms could have several clinical applications. Here, we concentrate on two areas. These are probing the mechanisms and effectiveness of ASMs and aiding with the localization of subtle epileptogenic focal abnormalities in surgical planning/evaluation.

#### Applying laminar fMRI to detect focal cortical dysplasia not visible via structural imaging

Drug-resistance affects approximately 40% of patients with focal epilepsy.^133,134^ In children, the most common cause is FCD, a localized malformation of cortical development.^135^ FCD is also a leading cause of medically refractory focal epilepsy in adults, often ranking as the second or third most common cause.^131,136^ Consequently, surgical resection of these lesions is a frequent and important treatment strategy.

The success of surgery relies on accurately identifying the seizure-generating lesion(s). Structural MRI is the primary tool for localizing FCD, but these lesions can be radiologically subtle. At conventional field strengths (1.5–3 T), around 30% of FCD cases are ‘MRI-negative’, meaning the lesion is not detectable on the images even for experienced clinicians.^137-139^ While the increased availability of 7 T MRI has improved detection rates, with a diagnostic gain of 22%–43% over lower field strengths,^140^ a substantial number of patients still have FCD lesions that cannot be identified with structural MRI alone. This poses a significant challenge for surgical planning and ultimately for achieving seizure control, potentially leading to the need for invasive methods such as iEEG to provide localization with obvious drawbacks.

This limitation highlights the need for complementary, non-invasive diagnostic tools that can improve the identification and characterization of FCD lesions. Laminar fMRI has the potential to help fill this gap by revealing the functional consequences of FCD, even when the structural abnormality is subtle or undetectable on conventional MRI. FCD, regardless of the specific subtype, disrupts the normal laminar organization of the cortex. This disruption manifests in various ways, including abnormal layering, the presence of dysmorphic neurons, and balloon cells. The International League against Epilepsy (ILAE) classifies FCD into three main types with subtypes^141^: type I, characterized by abnormal cortical layering, often including microcolumns (vertical columns of neurons) in type Ia; type II, distinguished by the presence of dysmorphic neurons, with balloon cells present in type IIb; and type III, defined by cortical dyslamination associated with another lesion (e.g. hippocampal sclerosis, tumour, vascular malformation).

Laminar fMRI’s ability to measure activity at distinct cortical depths makes it potentially well suited to detect the functional consequences of layer-specific disruptions caused by FCD (i.e. deep layers). Given that FCDs appear to initiate seizures from deep layers, markers of such activity could be used by researchers to identify abnormal activity across the brain to help identify FCD locations via markers of connectivity changes in these layers. Beyond identifying the epileptogenic zone, laminar fMRI could map the functional extent of the seizure network surrounding the FCD. By identifying regions with abnormal interictal layer-specific activity, it could assist surgeons in precisely delineating resection margins, maximizing the chances of successful seizure control while minimizing the removal of healthy tissue. Given the differences in FCD subtype functional motifs at the laminar scale could contain information relating to FCD subtype which would improve prognostication. Beyond pre-surgical planning, laminar fMRI could also contribute to post-surgical evaluation. Assessing changes in layer-specific activity after surgery could help monitor intervention effectiveness and identify any residual epileptogenic tissue, informing further treatment decisions and improving long-term seizure control. One unique feature of epilepsy in this context is that it affords the opportunity to link structural abnormalities in lamination and a host of molecular and genetic measures from surgically resected tissue to their functional output providing validation of the imaging markers and improving our understanding of these relationships.

In summary, by providing a more nuanced, layer-specific understanding of the functional impact of FCD in humans and its associated networks, laminar fMRI has the potential to significantly improve the accuracy of surgical planning and can be validated with post-surgical tissue evaluation, ultimately leading to more effective surgical interventions for patients with FCD.

#### Laminar fMRI in refining the knowledge of ASM mechanisms on epileptogenic networks

Much of the current understanding of the anticonvulsant effects of ASMs has emerged from the use of animal seizure and epilepsy models and *in vitro* cell and brain slice preparations.^6,142,143^ Findings from such studies have provided key insights into nano-scale, molecular seizure mechanisms and provided the basis for drugs that aim to target them. These studies have indicated that most available ASMs exert their actions through a modulatory effect on voltage- and receptor-gated ion channels strongly indicating that ion channel modulation is a significant, though likely not exclusive, mechanism of action. However, these models fail to capture the complexity of human epilepsy, including the range of seizure types, comorbidities and individual variability. Significant differences also exist between animals and humans in brain structure, organization and drug pharmacokinetics.^144-146^ Therefore, macro-scale pharmacological studies are essential to translate findings from animal models to human patients and to optimize ASM dosage and delivery.

Results from pharmaco-fMRI have revealed drug/network-specific activations for many commonly used ASMs.^147-149^ For example, studies in patients diagnosed with JME often report increased activation in motor areas, especially during motor tasks. In these patients, valproate appears to attenuate this excessive motor cortex co-activation.^150,151^ This suggests that valproate’s effectiveness in treating myoclonic jerks in JME might be related to its modulation of the motor network. Similarly, in patients diagnosed with focal epilepsy, studies using topiramate have shown reduced activation in language-related brain areas, such as the inferior frontal gyrus, during verbal fluency tasks.^152,153^ This reduced activation correlates with impaired performance on these tasks, suggesting that topiramate’s impact on the language network contributes to its known cognitive side effects, particularly difficulties with language and speech.

While the contribution of nano-scale animal model research to understanding epilepsy pathophysiology and developing treatments has been key, and macro-scale pharmaco-fMRI has provided valuable insights into ASM impact on network activity, a significant gap remains between these scales. Given that differing pathology at the nano-scale may lead to similar alterations in microcircuit function, ultimately contributing to seizure generation across networks, obtaining measurements at the intermediate, micro-scale within these networks is crucial. Laminar fMRI may provide a complementary, bridging level of analysis, offering additional data to refine our knowledge of ASM mechanisms in patients with epilepsy and the variability in treatment response. Laminar fMRI has several potential applications in this context, and a proof-of-concept study could investigate how lamotrigine (LTG) influences laminar activity within macro-scale networks.

#### Bridging the gap between the nano- and macro-scale effects of lamotrigine

LTG^154^ is currently used clinically as an anticonvulsant drug to treat focal seizures, primary and secondary tonic-clonic seizures, as well as seizures associated with Lennox-Gastaut syndrome.^155^ At the nano-scale, research has shown that LTG selectively binds and inhibits voltage-gated sodium channels, stabilizing presynaptic neuronal membranes and inhibiting presynaptic glutamate and aspartate release. In addition, while LTG’s primary mechanism of action is understood to be related to sodium channels, its precise effects are still being investigated. Although no significant impact on neurotransmitter systems such as serotonin, norepinephrine or dopamine has been definitively shown,^156^ there is a theory that LTG might also interact with calcium channels, which could explain its diverse therapeutic effects. These diverse molecular actions likely contribute to the network-scale effects of LTG.^154^

Previous pharmaco-fMRI studies have reported that LTG has widespread network effects.^157-159^ Consistent with these findings, Xiao *et al*.^157^ found that LTG-treated patients with drug-resistant focal epilepsy exhibited abnormal deactivations in areas of the DMN during a verbal fluency task, compared to patients on levetiracetam and healthy controls. This suggests that LTG may interfere with the normal suppression of the DMN during cognitive tasks. Of course, while providing understanding of the drug’s network scale effects, these findings are far removed from the modulation of sodium channels by which LTG is proposed to exert its primary anti-convulsant effects in patients with epilepsy. Crucially, there have also been some micro-scale findings that provide a basis for investigating LTG’s impact on the DMN at the micro level with laminar fMRI.

Lehnhoff *et al*.^160^ investigated the effects of LTG on the hyperpolarization-activated current (l_h_) in layer 2/3 neocortical pyramidal neurons from patients with focal epilepsy. Hyperpolarization-activated current, an inward current activated by hyperpolarization and generated by hyperpolarization-activated cyclic nucleotide-gated (HCN) channels, plays a critical role in regulating resting membrane potential, neuronal firing rate, rhythmicity and dendritic integration, thereby influencing overall brain activity.^161^ Dysfunction of HCN channels, and consequently (l_h_), has been implicated in several neurological disorders,^162,163^ including epilepsy, where its disruption can contribute to neuronal hyperexcitability and seizures.^164,165^ This *ex vivo* study, using tissue slices from patients with drug-resistant focal epilepsy (DRE), demonstrated that LTG enhances (l_h_) in layer 2/3 neurons. This enhancement reduces neuronal excitability by attenuating dendritic integration, making these neurons less responsive to excitatory inputs. While studies covered in this review indicate that many types of seizures initiate in deeper cortical layers, layer 2/3 is of particular interest because its neurons have extensive horizontal connections, making them important in propagating cortical activity. Therefore, the LTG-induced reduction in excitability within this layer is likely a crucial component of its antiseizure mechanism. Speculating, LTG may exert its therapeutic effect by preventing seizure propagation rather than initiation, contributing to the drug’s ability to dampen hyperexcitable networks and prevent seizure spread. This focus on layer 2/3 highlights the importance of understanding layer-specific drug effects in epilepsy treatment and suggests a potential target for laminar analysis of brain networks, such as the DMN, similar to Huber *et al*.^109^

By combining whole-brain coverage with layer-specific resolution, laminar fMRI can investigate how LTG modulates activity within specific layers of the DMN, a network implicated in LTG’s cognitive side effects. Following the work of Huber *et al*.,^109^ a proof-of-concept study could investigate the effects of LTG on the DMN using laminar fMRI. This study could examine changes in layer-specific functional connectivity within the DMN before and after LTG administration. Given the *ex vivo* findings of Lehnhoff *et al*.^160^ demonstrating LTG’s influence on layer 2/3 excitability, a laminar fMRI study could specifically test the hypothesis that LTG alters the functional connectivity of layer 2/3 within the DMN. For example, the study could investigate whether LTG reduces the functional coupling between layer 2/3 of key DMN nodes, such as the medial prefrontal cortex and the posterior cingulate cortex. Furthermore, by comparing the effects of LTG on different layers within the DMN, the study could reveal whether the drug’s influence is layer-specific, as suggested by the *ex vivo* data.

New layer-specific information could provide crucial insights into how LTG modulates network activity and how these modulations relate to its therapeutic effects and cognitive side effects. It is important to note this focus on layer 2/3, while consistent with the *ex vivo* findings on LTG’s effects on these neurons, appears to contrast with much of the *in vivo* animal data indicating that seizure initiation often occurs in deeper cortical layers. This apparent discrepancy highlights the complexity of epilepsy and the potential for distinct mechanisms to be involved in seizure initiation versus propagation, as well as the potential for drug effects to be layer- and even region-specific.

Laminar fMRI, by enabling the investigation of drug effects across the cortical depth, may help to reconcile these seemingly disparate findings and provide a more comprehensive understanding of how ASMs exert their therapeutic actions. By directly linking layer-specific changes in functional connectivity to the drug’s known molecular targets, laminar fMRI offers a powerful approach to refine our understanding of ASM mechanisms and potentially personalize treatment strategies for patients with epilepsy. This approach could be extended to other ASMs, providing a comprehensive understanding of how these drugs impact cortical microcircuits and ultimately leading to the development of more targeted and effective therapies.

## Summary of strengths and weaknesses

Laminar fMRI holds significant promise for advancing our understanding of epilepsy, offering the potential to bridge the gap between nano-scale molecular mechanisms and macro-scale network dysfunction. The evidence reviewed here strongly supports the notion that microcircuit and layer-specific dysfunction play a critical role in both seizure generation and propagation. Laminar fMRI, with its capacity to non-invasively probe cortical depths, offers an opportunity to translate these findings to human studies. The ability to visualize layer-specific activity and connectivity *in vivo* could improve our knowledge of epileptogenesis, seizure evolution, improving the location of FCDs and understanding the impact of ASMs in humans.

However, it is crucial to acknowledge that the direct correspondence between laminar fMRI signals and the detailed electrophysiological activity observed with microelectrodes remains, while plausible to some extent, speculative. Studies have demonstrated correlations between iEEG and fMRI activity in different layers^103^ and even linked iEEG with fMRI responses^104^; however, the spatial resolution, SNR and particularly temporal resolution of fMRI are still limited compared to electrophysiological recordings, and it may not be possible to detect subtle epileptogenic changes in the laminar fMRI signals. Therefore, further methodological development and validation studies will be needed to firmly establish the sensitivity and specificity of laminar fMRI for detecting layer-specific epileptic activity. Another important consideration is the relationship between interictal and ictal activity. While laminar fMRI may be sensitive to large signal changes during ictal events, it will not be possible to measure patients with epilepsy during most types of seizure for obvious technical reasons, and it is an open question as to whether it will be sufficiently sensitive to interictal activity.

Despite these limitations, we propose that the potential use of laminar fMRI in the study of epilepsy represents a viable approach to reconcile data across different scales in epilepsy research. While acknowledging the potential challenges of this application, we propose that laminar fMRI offers an opportunity to investigate the microcircuitry of epilepsy in the human brain, adding a new dimension to our understanding and treatment of epilepsy.

## Supplementary Material

awaf320_Supplementary_Data
