# Supplementary material for: The potential of laminar functional MRI in refining the understanding of epilepsy in humans
Source: Brain. 2025 Sep 3;148(12):4180–97. doi: 10.1093/brain/awaf320 (PMC12677026; doi:10.1093/brain/awaf320)
Supplement: awaf320_Supplementary_Data [file awaf320_supplementary_data.pdf]

Supplementary Table 1- Comparative Overview of Neurophysiological Modalities in Epilepsy Research

Modality	Primary measure	Typical spatial resolution	Typical temporal resolution	Key strengths	Key weaknesses
EEG (scalp)	Synchronous post-synaptic potentials	Centimetres	Milliseconds	Non-invasive, excellent temporal resolution, wide availability	Poor spatial localization, sensitive to artifacts
sEEG/ECoG (iEEG)	Direct neural activity (Local Field Potentials)	Millimetres	Milliseconds	High signal-to-noise, direct measure, good spatial/temporal for focal activity	Invasive, limited coverage, sampling bias
Microelectrode Arrays	Single-unit/multi-unit activity, LFPs	Micrometres	Milliseconds	Cellular/microcircuit level detail, highly localized	Highly invasive, very limited coverage, single-patient research
Conventional fMRI	BOLD response (hemodynamic activity)	Millimetres	Seconds	Whole-brain coverage, non-invasive, structural/functional maps	Indirect measure, limited temporal resolution, sensitive to motion
Laminar fMRI	Layer-specific BOLD response	Sub-millimetre	Seconds	Layer-specific insights, non-invasive, bridges scales	As conventional fMRI but even more sensitive to motion, technical challenges, limited clinical availability.
